# Dietary supplementation of hemp oil in teddy dogs: Effect on apparent nutrient digestibility, blood biochemistry and metabolomics

**DOI:** 10.1080/21655979.2022.2043018

**Published:** 2022-02-24

**Authors:** Guosheng Xin, Jie Yang, Ruiguo Li, Qiaoxian Gao, Ronglin Li, Jianguo Wang, Juan Zhang, Jing Wang

**Affiliations:** a School of Life Science, Ningxia University, Yinchuan, Ningxia Hui Autonomous, China; b Ningxia Feed Engineering Technology Research Center, Ningxia University, Yinchuan, Ningxia Hui Autonomous, China; c Petpal Pet Nutrition Technology Co., Ltd, Hangzhou, Zhejiang province, China; d School of Agriculture, Ningxia University, Yinchuan, Ningxia Hui Autonomous Region, China; e Ningxia Hiby Analysis & Testing Institute, Yinchuan, Ningxia Hui Autonomous, China

**Keywords:** Hemp oil, dog, nutrient digestibility, blood biochemistry, metabolomics

## Abstract

Present study aimed to evaluate the influence of distinct concentration of dietary supplements hemp oil on apparent nutrient digestibility, blood biochemical parameters and metabolomics of teddy dogs. A total of 25 healthy teddy dogs were selected and divided into five treatments according to diet supplements hemp oil at a rate of 0% (A), 0.5% (B), 1% (C), 2% (D), and 4% (E). Appropriate added hemp oil improved apparent nutrient digestibility of dry matter, crude protein and crude fat (86.32–88.08%, 86.87–88.87% and 96.76–97.43%). The hemp oil significantly increased blood biochemical of utilization related total protein, albumin and globulin (61.33–69.54, 35.08–40.38 and 26.53–31.63 g/L), immunity capacity related immunoglobulin E and γ-interferon (203–347kU/L and 23.04–25.78ng/L), energy-related thyroxine and triiodothyronine (27.11–36.75 and 0.94–1.67 nmol/L). In addition, hemp oil improved superoxide dismutation (26.47–33.02 U/ml) and reduced malondialdehyde (5.30–3.28 nmol/ml). The differential metabolites mainly included nucleotides and metabolites of oxidized lipids, bile and other fatty acids, coenzymes and vitamins. The main metabolic pathways included purine and arachidonic acid metabolism, bile and unsaturated fatty acid biosynthesis, cell oxidative phosphorylation and rheumatoid arthritis. Overall, appropriate dietary supplements hemp oil positively to nutrient digestibility and blood metabolism, immunity and antioxidant capacity, 1% to 2% hemp oil supplements was recommended for teddy dog diet.

## Graphical abstract

**Figure:**
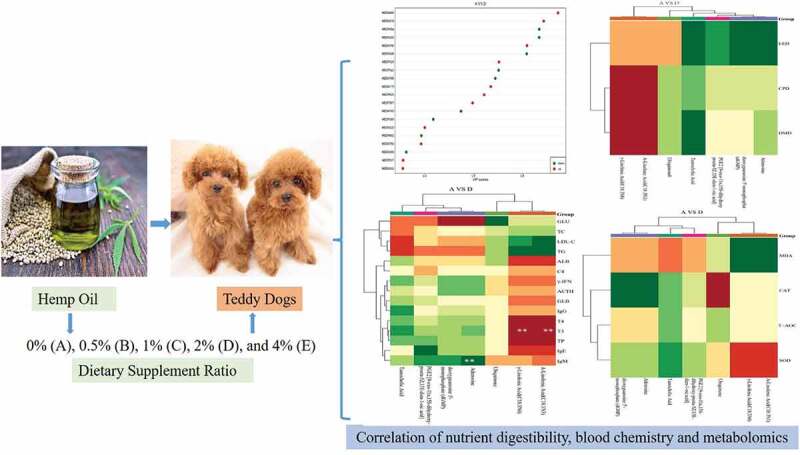

## 1. Introduction

Pets have become an important part of life with the improvement of living standards. It is estimated that approximately 94.2 million pet dogs in the United States, 50–60 million pet dogs (70% of total pets) in China and teddy are the most popular small dog (18% of total pet dogs) [1]. Therefore, aspect of animal nutrition, there is a great requirement for sufficient pet foods with good production capacity and better nutritional characteristics [2]. At present, development of additives in animal nutrition attracted wide attention to fulfill the adequate nutrients [3], such as dietary supplement organically chelated copper [4], microencapsulated phytogenic [5], oil [2] and krill meal [6] for dogs. Among them, hemp oil is considered as a beneficial fatty acid resource due to its high content of polyunsaturated fatty acid (80%) [7]. Specifically, a 3:1 ratio of ω-6 linoleic to ω-3 alpha-linolenic acid was recommended for a healthy diet, which is better than other vegetable oils such as olive, soybean, rapeseed and peanut oil. Additionally, ingredients of γ-linolenic and stearidonic acid, fat-soluble vitamins D and E also contributed to a higher nutritional value of hemp oil [8,9].

Nutrient digestibility directly reflects the feed nutritional value and influences blood parameters [10]. Studies have found that the addition of unsaturated fatty acids in diets improves the apparent digestibility of animal nutrients [11]. Papadomichelakis et al. [12] added soybean oil with high unsaturated fatty acids to rabbit diets and shown the significantly improved apparent digestibility of protein, crude fat and energy (P < 0.05). While Sabchuk et al. [2] observed that the supplements of corn oil in dog diet hadn’t significant effect on the apparent nutrient digestibility. Additionally, polyunsaturated fatty acids (PUFA) could improve the body’s antioxidant and immune capacity, further promote body metabolism [13]. Yu et al. [14] showed that appropriate amount of unsaturated fatty acids added in feed improves the antioxidant enzyme activity and antioxidant capacity, enhances immunity and improves growth performance of sea cucumber. Rezapour-Firouzi et al. [15] has shown that dietary n-6/n-3 PUFA ratio of 3:1 or 6:1 improves the serum immune antibody level, antioxidant enzyme activity, and the ability to remove lipid peroxides. Singer et al. [16] also showed that PUFA enhanced immunity and hemp oil increased the body’s superoxide dismutase and glutathione peroxidase as well as reduced the malondialdehyde level. Bontempo et al. [17] reported that dietary conjugated linoleic acid increased the immunoglobulin content and immunity of pigs. Jing et al. [18] pointed out that PUFA regulates the metabolism of the body’s substances by increasing the body’s insulin, serum growth hormone and thyroid hormone levels. Therefore, appropriate supplements PUFA in the daily diet is beneficial to promote nutrient digestibility, immunity and animal growth.

Metabolomics studied the metabolic profile and functional regulation of the whole organism by qualitative and quantitative analysis, which provide a unique perspective for evaluate diet-related comprehensive metabolism [19,20]. Till now, widely metabolomics apply to characterize nutrient metabolism and blood metabolites which directly characterized metabolic pathways associated with dietary intervention [21]. For instance, Wei et al. [22] applied metabolomics in aquaculture nutrition study to assess the nutritional requirements and dietary nutrients of aquatic species. However, there are few scientifically tested on hemp oil as pet dietary supplements and effect on nutritional utilization and blood metabolomics, which is essential for novel pet food ingredients safely and effectively. Therefore, this study dietary supplements hemp oil for teddy dogs and evaluates the apparent nutrient digestibility and blood biochemical and metabolomics, expected to obtain the optimal amount of dietary supplements addition and contribute to the resource utilization of hemp oil as a high-quality and reliable pet food.

## 2. Materials and methods

### 2.1. Experimental design and sample collection

The hemp oil provided from Bama Yishutang Health and Longevity Industry Co., Ltd (Guangxi, China) and the fatty acid composition is shown in Table 1. The test animal of twenty-five female teddy dogs aged 1–2 years were selected and divided into 5 groups with 5 replicates per group according to the similar body weight (4.75 kg ± 1.34 kg) and gender consistency principle. All dogs received clinical and physical examinations as well as pre-vaccinated and dewormed. The supplementation level of hemp oil in the diet was 0% (A as control), 0.5% (B), 1% (C), 2% (D) and 4% (E). The trial lasted for 45 days including 15 days pre-feeding and 30 days regular feeding. The diet was provided in line with Nutrition Requirements for Dogs and Cats [23] to fulfill the daily metabolic energy requirements of adult pet dogs, and the ingredients presented in Table 2. The total feeding amount was calculated according to the formula ME (kcal/d) = 140 × BW^0.75^ (ME and BW was measured energy and metabolic weight) and all dogs were feed at 08:00 and 16:00 every day. During the feeding and management process in Ningxia Pet Farm, the kennel was immunologically disinfected in accordance with conventional methods. The test dogs were fed in single cages and the feeding, drinking, diarrhea, death and elimination were observed in detail.

**Table 1.** Fatty acid composition of hemp seed oil

Fatty acid composition		Content %
C4:0	Butyrate	0.05
C14:0	Myristic acid	0.03
C15:0	Pentadecanoic acid	0.23
C16:0	Palmitic acid	6.33
C16:1	Palmitoleic acid	0.09
C17:0	Margaric	0.05
C17:1	Margaroleic	0.03
C18:0	Stearic	2.88
C18:1n9t	Trans-elaidic	0.00
C18:1n9c	Oleic	13.05
C18:2n6c	Linoleic	58.71
C20:0	Arachidic	0.69
C18:3n6	γ-Linolenic	0.67
C18:3n3	α-Linolenic	16.81
C20:2	Eicosadienoic	0.05
C22:0	Behenic	0.24
C24:0	Lignoceric	0.08
UFA Unsaturated fatty acid		89.42
SFA Saturated fatty acid		10.58

**Table 2.** Composition and nutrient level of experimental diets (Dry matter basis)

Items	Content %				
	A	B	C	D	E
Ingredients					
Chicken bone meal	10.20	10.21	10.24	10.27	10.29
Poultry by-product meal	15.01	14.99	14.97	14.93	14.92
Chicken liver powder	1.00	1.00	1.00	1.00	1.00
Corn flour	18.95	18.96	19.09	19.12	19.13
Wheat	20.04	20.02	19.86	19.78	19.68
Oat	22.99	23.00	23.01	23.05	23.10
Yeast extract	1.00	1.00	1.00	1.00	1.00
Chicken oil	9.81	9.32	8.83	7.85	5.88
Hemp seed oil	0.00	0.50	1.00	2.00	4.00
2% Premix 2%^1)^	1.00	1.00	1.00	1.00	1.00
Total	100.00	100.00	100.00	100.00	100.00
Nutrient levels^2)^					
CP	27.49	27.49	27.49	27.49	27.49
EE	14.36	14.36	14.35	14.35	14.35
CF	2.17	2.17	2.17	2.16	2.16
Ash	3.43	3.43	3.43	3.43	3.43
Ca	0.93	0.93	0.93	0.93	0.93
P	0.76	0.76	0.76	0.76	0.76
ME (Kcal/Kg)	4023.38	4023.37	4023.43	4023.45	4023.32

CP: crude protein, EE: crude fat, CF: crude fiber, Ca: Calcium, P: Phosphorus, ME: Metabolizable energy. A: dietary supplements 0% hemp oil in teddy dog (as control), B: dietary supplements 0.5% hemp oil in teddy dog, C: dietary supplements 1% hemp oil in teddy dog, D: dietary supplements 2% hemp oil in teddy dog and E: dietary supplements 4% hemp oil in teddy dog.

The fecal samples were collected according to total feces collection method and recorded the weight one week before the end of the experiment. Two sets of samples were collected from each dog, one part was placed in a cryotube at −80°C and another part was added with 2% sulfuric acid and stored in refrigerator at −20°C. In addition, 10 mL blood sample was collected from the fasting forearm vein of dogs and kept in a vacuum blood collection tube one day before the end of the experiment. After standing for 3 hours, the blood was centrifuged at 3000 rpm/min for 15 min, then prepared the serum and stored in liquid nitrogen for analysis.

### 2.2. Determination of apparent nutrient digestibility and blood indexes

For the apparent nutrient digestibility determination, fecal samples were dried at 105°C and crushed after cooling then passed through a 40-mesh sieve, the diet and pretreated fecal dry matter (DM), crude protein (CP), crude fat (EE) and energy were determined according to Direkvandi et al. [10]. The apparent nutrient digestibility was calculated according to formula:

Nutrient apparent digestibility (%) = [(Nutrient intake – Nutrient excretion)/Nutrient intake] × 100.

For blood analysis, prepared serum was sent to Ningxia Di’an Lejia Medical Inspection Center (Co., Ltd.) to determine the biochemical indexes. There was mainly included immunoglobulin M (IgM), immunoglobulin G (IgG), immunoglobulin E (IgE), complement 4 (C4), γ-interferon (γ-IFN), total protein (TP), albumin (ALB), globulin (GLB), thyroxine (T4), triiodothyronine (T3), adrenocorticotropic hormone (ACTH), triglycerides (TG), total cholesterol (TC), low-density lipoprotein cholesterol (LDL-C) and blood sugar (GLU). Additionally, the prepared serum was sent to Ningxia Hiby Analysis & Testing Institute to determine the serum antioxidant indexes, mainly comprised of superoxide dismutase (SOD), total antioxidant capacity (T-AOC), catalase (CAT), and malondialdehyde (MDA). Furthermore, the serum samples were thawed on the ice and vortexed mix 10s, then 50 uL sample put into EP tube and added 150 uL precooled ice methanol (contained 1 ug/mL 2-chlorophenylalanine as internal standard). Thereafter, vortex for 3 min at 12,000 r/min and centrifuged 10 min at 4°C, then transferred the supernatant into a new EP tube, further centrifuged at 12,000 r/min for 5 min, and then transferred supernatant to the sample bottle for liquid chromatograph-mass spectrometer and tandem mass spectrometry (LC-MS/MS) analysis.

### 2.3. Data processing

All data first arranged by using Excel 2016, then performed one-way analysis of variance and significance analysis by SPSS 25.0. The Duncan method in one-way ANOVA was used for statistical analysis and the significance level was P < 0.05. All the results were present as a form of mean ± standard deviation.

## 3. Results and discussion

### 3.1. Dietary supplements hemp oil effect on nutrient digestibility and blood biochemistry of teddy dogs

The apparent nutrient digestibility of dry matter, crude protein and energy were improved with the increased supplements of hemp oil from 0% to 2% (86.32–88.08%, 86.87–88.87% and 96.76–97.43% respectively), while suppressed in 4% hemp oil dietary supplementation. Notably, 1% and 2% hemp oil added treatments has higher dry matter digestibility (87.43% and 88.08%) and crude protein digestibility (88.77% and 88.87%), while 0.5% and 4% hemp oil supplements had higher crude fat digestibility (97.50%) and energy digestibility (91.35%) (Table 3). Thus, different amount of hemp oil dietary supplements varied in digestibility performances and better in 1–2% hemp oil addition. Kholif et al. [24] also found that the addition of crude coriander oil in the diet of goats improves the apparent energy digestibility, Kim et al. [25] found that the addition of delta-aminolevulinic acid in diet improves the apparent nutrient digestibility and growth of weaned pigs. However, the difference in digestibility between treatments was not significant (P > 0.05), which was different with Barszcz et al. [26] and might be related to the source of unsaturated fatty acids and the differences in experimental animals.

**Table 3.** The effect of hemp oil on the apparent nutrient digestibility of teddy dogs

Index	Treatments				
	A	B	C	D	E
Apparent Digestibility of Dry Matter (DMD %)	86.32 ± 1.37	87.02 ± 0.83	87.43 ± 1.36	88.08 ± 1.71	87.03 ± 2.99
Apparent digestibility of crude protein (CPD %)	86.87 ± 2.04	88.27 ± 0.56	88.77 ± 1.15	88.87 ± 2.01	88.71 ± 1.82
Apparent digestibility of crude fat (EED %)	96.76 ± 1.11	97.50 ± 0.37	96.35 ± 1.02	97.43 ± 0.86	96.92 ± 0.69

A: dietary supplements 0% hemp oil in teddy dog (as control), B: dietary supplements 0.5% hemp oil in teddy dog, C: dietary supplements 1% hemp oil in teddy dog, D: dietary supplements 2% hemp oil in teddy dog and E: dietary supplements 4% hemp oil in teddy dog.

Numerous studies have shown that PUFA improves body immunity and promotes animal growth, also reduces thrombosis and incidence of cardiovascular diseases by adjusted blood biochemistry [27]. After dietary supplements hemp oil, the content of TP, ALB and GLB were increased (61.33–69.54, 35.08–40.38 and 26.53–31.63 g/L respectively), which indicates the strengthen ability of utilize protein and conducive to feed conversion [28]. Appropriate added hemp oil in dog diets positively improved the immunity capacity (related with IgG, IgE, IgM, and γ-IFN) while inhibited after excessive addition (4%) (Table 4). Hughes et al. [29] shown that n-3PUFA inhibit the mitotic response of the T cells and spleen cells, high levels of n-3PUFA in diets also reduced immunity capacity. Baira et al. [20] also confirmed that the addition of PUFA in chicken diets could improve the humoral immune function, but reduced in excessive addition. Finally, appropriate amount addition of hemp oil positively affects the protein absorption and immunocompetence.

**Table 4.** The effect of hemp oil on dog blood biochemical indexes

Parameters	Treatments				
	A	B	C	D	E
IgM (g/L)	0.88 ± 0.45^b^	1.07 ± 0.32^ab^	1.39 ± 0.12^a^	1.52 ± 0.12^a^	1.49 ± 0.12^a^
IgG (g/L)	4.00 ± 0.83	4.00 ± 0.65	4.38 ± 0.44	4.46 ± 0.40	4.49 ± 0.40
IgE (kU/L)	203.60 ± 83.42	321.63 ± 89.62	321.67 ± 58.10	347.88 ± 78.97	246.08 ± 33.74
C4 (g/L)	0.06 ± 0.01	0.08 ± 0.02	0.08 ± 0.01	0.08 ± 0.01	0.07 ± 0.01
γ-IFN (ng/L)	23.04 ± 5.14^b^	24.63 ± 6.78^b^	31.32 ± 5.73^a^	31.78 ± 9.30^a^	25.78 ± 6.64^b^
TP (g/L)	61.33 ± 2.97^b^	68.10 ± 3.04^a^	69.54 ± 4.13^a^	69.30 ± 2.73^a^	65.60 ± 4.25^ab^
ALB (g/L)	35.08 ± 3.90^b^	38.76 ± 2.58^a^	39.20 ± 2.39^a^	38.66 ± 0.43^a^	40.38 ± 2.06^a^
GLB (g/L)	26.53 ± 3.18	27.93 ± 1.42	31.63 ± 2.50	29.78 ± 3.00	27.48 ± 2.93
T4 (nmol/L)	27.11 ± 3.45^b^	31.30 ± 3.93^ab^	32.23 ± 4.60^ab^	36.75 ± 2.91^a^	33.98 ± 4.11^a^
T3 (nmol/L)	0.94 ± 0.07^b^	1.24 ± 0.15^ab^	1.67 ± 0.51^a^	1.50 ± 0.17^a^	1.49 ± 0.16^a^
ACTH (pg/ml)	7.80 ± 0.45^b^	8.24 ± 1.49^b^	8.41 ± 1.80^b^	9.20 ± 1.89^ab^	11.41 ± 0.78^a^
TG (mmol/L)	0.76 ± 0.06^a^	0.75 ± 0.17^a^	0.47 ± 0.08^b^	0.53 ± 0.06^b^	0.55 ± 0.16^b^
TC (mmol/L)	5.55 ± 1.46	4.83 ± 0.31	4.53 ± 1.25	4.58 ± 1.35	4.55 ± 1.35
LDL-C (mmol/L)	1.49 ± 1.24^a^	1.23 ± 0.55^a^	0.34 ± 0.16^b^	0.26 ± 0.08^b^	0.31 ± 0.06^b^
GLU (mmol/L)	5.03 ± 0.58	4.76 ± 0.65	4.65 ± 0.15	4.57 ± 0.24	4.62 ± 0.24

IgM: immunoglobulin M, IgG: immunoglobulin G, IgE: immunoglobulin E, C4: complement 4, γ-IFN: γ-interferon, TP: total protein, ALB: albumin, GLB: globulin, T4: thyroxine, T3: triiodothyronine, ACTH: adrenocorticotropic hormone, TG: triglycerides, TC: total cholesterol, LDL-C: low-density lipoprotein cholesterol, GLU: blood sugar. A: dietary supplements 0% hemp oil in teddy dog (as control), B: dietary supplements 0.5% hemp oil in teddy dog, C: dietary supplements 1% hemp oil in teddy dog, D: dietary supplements 2% hemp oil in teddy dog and E: dietary supplements 4% hemp oil in teddy dog.

The thyroid hormone (T4) and triiodothyronine (T3) plays an essential role in protein synthesis, temperature regulation and energy production [30]. The content of T4 and T3 increased to 27.11–36.75 and 0.94–1.67 nmol/L after hemp oil supplements. The adrenocorticotropic hormone (ACTH) active in reproductive organ growth, differentiation and maintenance of fertility [31], the content increased from 7.80 to 11.41 pg/mL in hemp oil added treatments. In addition, the concentration of TC and TG reflects the lipid metabolism, and the LDLC mainly transports cholesterol from the liver to extrahepatic tissues [32]. Hemp oil supplements significantly reduced the content of TG and LDLC (P < 0.05) and lowest in treatment C and D (0.47 and 0.26 mmol/L). Might be due to PUFA inhibited fatty acid synthase (FAS) and diglyceride transacylase (DGAT) and the activity of hydroxymethylglutarate-coenzyme A (HMG-COA) reductase, promoted the fatty acid oxidative decomposition and down-regulated LDL receptors and inhibits TC synthesis and absorption, thereby reduce the TG and TC content [33,34]. Studies have shown that PUFA effectively reduced blood total cholesterol, triglycerides, and low-density lipoprotein cholesterol in humans and animals, and regulated lipid metabolism and prevented cardiovascular and cerebrovascular diseases [35,36].

The antioxidant index of superoxide dismutase (SOD) has a strong effect on scavenge active oxygen and free radicals, which improve the body’s antioxidant capacity [37]. The SOD activity in serum was significantly increased (P < 0.05) after dietary supplement of hemp oil, and highest in E (33.02 U/mL). In addition, total antioxidant capacity (TAO) represents the antioxidant capacity of the body’s defense system and catalase (CAT) provides the antioxidant defense and protection [26]. The content of TAO and CAT was increased with hemp oil supplements (0.38–0.40 mM and 3.72–4.23 U/ml) and highest in treatment C (0.40 mM and 4.23 U/ml). The malondialdehyde (MDA) produced by lipid peroxide catabolism and evaluated the lipid peroxidation degree [24], while the MDA content was significantly reduced (P < 0.05) after hemp oil addition and lowest in treatment D (3.28 nmol/ml) (Table 5). Dietary supplement appropriate amount of PUFA significantly promote the fat metabolism and growth performance, but excessively high levels of PUFA caused lipid peroxidation and oxidative stress further increased in malondialdehyde [38]. Overall, hemp oil positively to antioxidant capacity and 2% addition demonstrated better performance.

**Table 5.** The effect of hemp oil on the anti-oxidation indexes of dog serum

Parameters	Treatments				
	A	B	C	D	E
SOD (U/mL)	26.47 ± 2.65^c^	27.83 ± 2.94^bc^	28.99 ± 1.69^bc^	30.63 ± 1.29 ^ba^	33.02 ± 3.25^a^
T-AO (mM)	0.38 ± 0.00	0.39 ± 0.02	0.40 ± 0.02	0.38 ± 0.05	0.39 ± 0.04
CAT (U/ml)	3.72 ± 0.43	4.08 ± 0.62	4.23 ± 0.54	4.20 ± 1.10	3.84 ± 0.38
MDA (nmol/ml)	5.30 ± 1.04^a^	4.85 ± 1.02^a^	3.83 ± 0.39^ab^	3.28 ± 0.71^b^	4.95 ± 1.13^a^

SOD: superoxide dismutase, T-AO: total antioxidant capacity, CAT: catalase, MDA: malondialdehyde. A: dietary supplements 0% hemp oil in teddy dog (as control), B: dietary supplements 0.5% hemp oil in teddy dog, C: dietary supplements 1% hemp oil in teddy dog, D: dietary supplements 2% hemp oil in teddy dog and E: dietary supplements 4% hemp oil in teddy dog.

### 3.2. Dietary supplements hemp oil effect on blood metabolomics of teddy dogs

**Figure 1.**
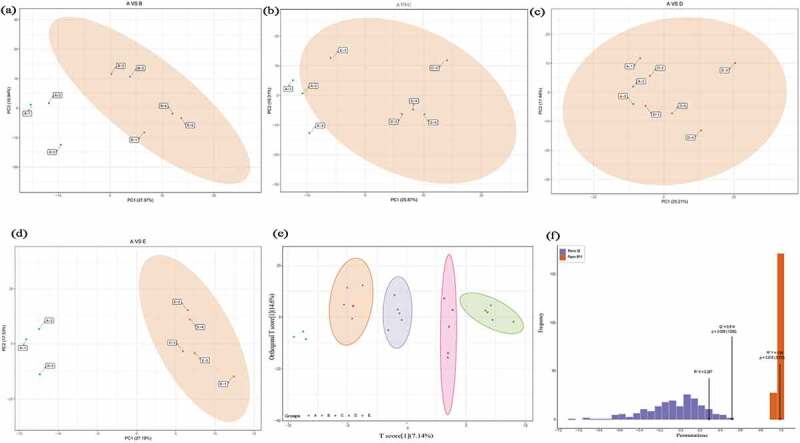
The principal component analysis (PCA) between control A and treatment B (Figure 1a), C (Figure 1b), D (Figure 1c), E (Figure 1d), and the orthogonal partial least square discriminant analysis (OPLS-DA) score (Figure 1e) and model verification (Figure f). The abscissa represents the model accuracy and the ordinate represents the frequency of model classification effect. The prediction parameters R^2^X and R^2^Y represent the interpretation rate of X and Y matrix, and Q^2^ represents the prediction ability of the model. When Q^2^ > 0.5 means effective model, and when Q^2^ > 0.9 represents excellent model.

Metabolomics data was characterized by ‘high-dimensional and massive’, combined with univariate and multivariate statistical analysis could accurately mined differential metabolites [39]. The principal component analysis (PCA) was carried out between control A and treatment B, C, D, and E (Figure 1a-d). The metabolic distribution between 0.5% and 4% hemp oil added treatments and control were well separated, and each treatment with 5 points were concentrated together, which indicated the metabolite modulation by hemp oil and reliability of the data. Thereafter, performed orthogonal partial least square discriminant analysis (OPLS-DA) and model verification (Figure 1e-f). Total 200 random permutation and combination data were conducted to verify the model. As shown in Figure 1e, the metabolite distribution clearly separated according to the different amount of hemp oil addition, the Q^2^ = 0.514 (P = 0.005) and R^2^Y = 0.99 (P = 0.015) indicated that the OPLS-DA model established was statistically significant, effective and reliable, which can be used for further metabolites screening (Figure 1f).

**Figure 2.**
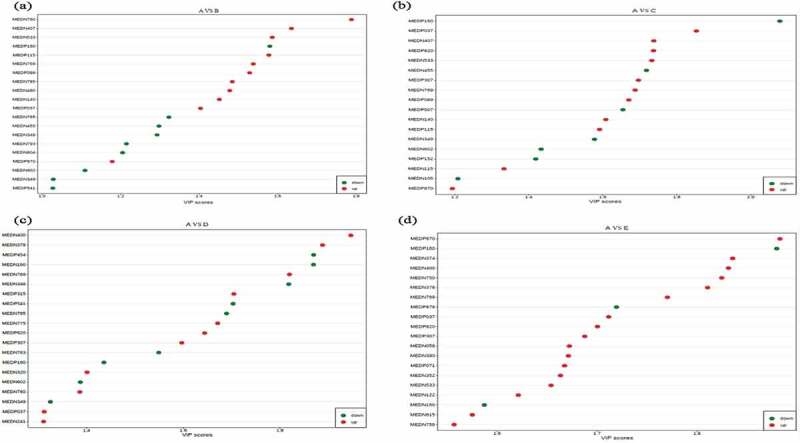
The statistical of differential metabolites-based VIP value between control A and treatment B (Figure 2a), C (Figure 2b), D (Figure 2c), and E (Figure 2d).

**Figure 3.**
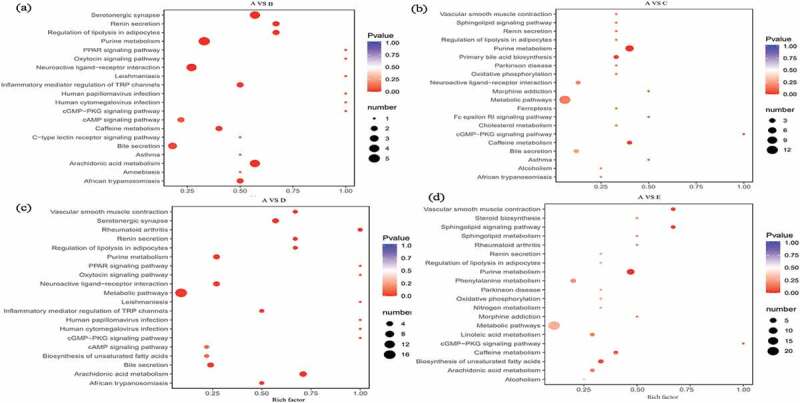
The pathway analysis of the detected differential metabolites between control A and treatment B (Figure 3a), C (Figure 3b), D (Figure 3c), and E (Figure 3d).

As perspective of screening differential metabolites, combine the variable importance in the projection (VIP) value of the OPLS-PA model (VIP ≥ 1) and the P-value of univariate analysis or difference multiple value (Fold Change FC ≥ 2 or FC ≤ 0.5) to obtain the potential biomarkers with significant difference. From the statistical of differential metabolites (Table 6) and VIP value (Figure 2) observation, a total of 20, 18, 27 and 43 significantly differential metabolites were screened out in treatment B, C, D and E, with 9, 7, 12, 9 down-regulated and 11, 11, 15, 34 up-regulated metabolites compared with control respectively. Furthermore, annotated the differential metabolites by kyoto encyclopedia of genes and genomes (KEGG) database and performed pathway analysis (Table 7 and Figure 3).

**Table 6.** Statistics of the different metabolites number

Treatments	Total Sig Metabolites	Down regulated	Up regulated
A_VS_B	20	9	11
A_VS_C	18	7	11
A_VS_D	27	12	15
A_VS_E	43	9	34

A: dietary supplements 0% hemp oil in teddy dog (as control), B: dietary supplements 0.5% hemp oil in teddy dog, C: dietary supplements 1% hemp oil in teddy dog, D: dietary supplements 2% hemp oil in teddy dog and E: dietary supplements 4% hemp oil in teddy dog.

**Table 7.** Differential metabolite Kyoto encyclopedia of genes and genomes (KEGG) pathway enrichment analysis

Treatments	Compounds	VIP	FC	Type	Pathway ID
A VS B	^9α,11,15S-^^trihydroxythromba−5Z,^^13E-^^dien−1^^−^^oic acid^	1.29	0.37	down	ko00590, ko04726
	^9α,15S-^^dihydroxy−11^^−^^oxo^^−^^prosta−5Z,^^13E-^^dien−1^^−^^oic acid^	1.03	0.31	down	ko00590, ko04080, ko04726, ko05143
	Deoxyguanosine5’-monophosphate (d GMP)	1.11	0.11	down	ko00230
	^5S,12R-^^dihydroxy−6Z,^^8E,10E,14Z-^^eicosatetraenoic^^acid^	1.48	4.79	up	ko00590, ko04080, ko04726, ko04750
	Adenosine	1.58	0.03	down	ko00230, ko04080, ko04923, ko04924
A VS C	Taurocholic Acid	1.21	0.14	down	ko00120, ko04976
	Chenodeoxycholic Acid	1.33	2.6	up	ko00120, ko04976
	^9α,15S-^^dihydroxy−11^^−^^oxo^^−^^prosta−5Z,^^13E-^^dien−1^^−^^oic acid^	1.58	0.44	down	ko00590, ko04080, ko04726
	Guanine	1.72	0.21	down	ko00230
	Deoxyguanosine5’-monophosphate (d GMP)	1.43	0.08	down	ko00230
	3’-Aenylic Acid	1.42	0.06	down	ko00230
	Ubiquinone	2.07	2.44	up	ko00130, ko00190
	Adenosine	2.08	0	down	ko00230
A VS D	Taurocholic Acid	1.1	0.2	down	ko04976
	Vitamin D3	1.31	2049.29	up	ko05323
	^9α,11,15S-^^trihydroxythromba−5Z,^^13E-^^dien−1^^−^^oic acid^	1.82	0.28	down	ko00590, ko04726, ko04976
	^9α,15S-^^dihydroxy−11^^−^^oxo^^−^^prosta−5Z,^^13E-^^dien−1^^−^^oic acid^	1.33	0.25	down	ko00590, ko04080, ko04726
	Deoxyguanosine5’-monophosphate (d GMP)	1.39	0.24	down	ko00230
	γ-Linolenic Acid (C18:3N6)	1.89	2.78	up	ko00591, ko01040
	A-Linolenic Acid (C18:3N3)	1.95	3.04	up	ko00592, ko01040,
	Ubiquinone	1.03	1733.93	up	ko00130, ko00190
	^9-^^oxo−11α,15S-^^dihydroxy^^−^^prosta−5Z,^^13E-^^dien−1^^−^^oic acid^	1.55	0.38	down	ko00590, ko04080, ko04726, ko04750, ko04923, ko04924, ko05323
	Adenosine	1.44	0.10	down	ko00230, ko04080, ko04270, ko04923, ko04924
A VS E	Guanosine	1.59	0.4	down	ko00230
	Ubiquinone	1.03	1733.93	up	ko00130, ko00190
	Deoxyguanosine 5’-monophosphate (d GMP)	1.34	0.07	down	ko00230
	^20-^^hydroxy−5Z,^^8Z,11Z,14Z-^^eicosatetraenoic^^acid^	1.38	2.58	up	ko04270
	3’-Aenylic Acid	1.53	0.1	down	ko00230
	Adenosine	1.88	0	down	ko00230, ko0407, ko04270

VIP: Variable importance projection, FC: Multiple of difference. A: dietary supplements 0% hemp oil in teddy dog (as control), B: dietary supplements 0.5% hemp oil in teddy dog, C: dietary supplements 1% hemp oil in teddy dog, D: dietary supplements 2% hemp oil in teddy dog and E: dietary supplements 4% hemp oil in teddy dog.

Compared with treatment B and control A, total 5 metabolites annotated by KEGG with up-regulated of leukotriene B4, and down-regulated of thromboxane B2 and prostaglandin D2, 2-’deoxyguanosine 5’-monophosphate, and adenosine. The main metabolic pathways involved in ko00590 (arachidonic acid metabolism), ko04726 (serotonergic synapse), ko00230 (purine metabolism), ko04080 (neuroactive ligand–receptor interaction), ko04924 (renin secretion), ko04923 (regulation of lipolysis in fat cells), ko04750 (regulation of inflammatory mediators of the TRP channel), and ko05143 (African trypanosomiasis). Compared with treatment C and control A, a total of 8 metabolites with corresponding KEGG annotations were screened. The up-regulated metabolites were chenodeoxycholic acid and ubiquinone, and the down-regulated metabolites were taurocholic acid, prostaglandin D2, guanine, 2- ‘Deoxyguanosine 5’-monophosphate, adenosine-3’-phosphate, and adenosine. The metabolic pathways related with ko00230 (purine metabolism), ko04976 (bile secretion), ko00120 (first-level bile acid biosynthesis), ko00590 (arachidonic acid metabolism), ko04726 (serotonergic synapse), ko04080 (neuroactive ligand–receptor interaction), ko00130 (biosynthesis of ubiquinone and other terpene quinones), and ko00190 (oxidized phosphoric acid change). Compared with treatment D and control A, a total of 10 metabolites with corresponding KEGG annotations were screened out and the up-regulated metabolites were vitamin D3, α-linolenic acid/octadecatrienoic acid (cis 9,12,15), γ-linseed acid (C18:3) and ubiquinone, down-regulated metabolites were taurocholic acid, thromboxane B2, prostaglandin D2, 2-’deoxyguanosine 5’-monophosphate (dGMP), prostaglandin E2 and adenosine. The related metabolic pathways were ko00590 (arachidonic acid metabolism), ko04726 (serotonergic synapse), ko05323 (rheumatoid arthritis), ko04080 (neuroactive ligand–receptor interaction), ko00230 (purine metabolism), ko00591 (linoleic acid metabolism), ko00592 (α-linolenic acid metabolism), ko00130 (biosynthesis of ubiquinone and other terpene quinones), ko04976 (bile secretion), ko00190 (oxidative phosphorylation) and ko01040 (unsaturated fatty acid biosynthesis).

Compared with treatment E and control A, a total of 6 metabolites with corresponding KEGG annotations were screened out and the up-regulated metabolites were ubiquinone and 20-hydroxy-5Z, 8Z, 11Z, 14Z-eicosatetraenoic acid, and down-regulated metabolites were guanosine, 2-’deoxyguanosine 5’-monophosphate, adenosine-3’-phosphate and adenosine. The metabolic pathways involved in ko00230 (purine metabolism), ko04071 (sphingolipid signaling pathway), ko04270 (contraction of vascular smooth muscle), ko00130 (biosynthesis of ubiquinone and other terpene quinones) and ko00190 (oxidative phosphorylation). Overall, a total of 440 metabolites were detected in dog serum and increased with the elevated amount of hemp oil supplements. The differential metabolites mainly included nucleotides and their metabolites of oxidized lipids, bile acids, lipids and other fatty acids, coenzymes and vitamins. As intermediate metabolites and substrates, participate in a variety of in vivo metabolism and the main metabolic pathways included purine metabolism, arachidonic acid metabolism, bile acid biosynthesis, unsaturated fatty acid biosynthesis, cell oxidative phosphorylation and rheumatoid arthritis.

Among them, the final product of purine metabolism was uric acid [24] and the significantly down-regulated of guanine, adenosine, guanosine, adenosine 3 phosphate, and 2-’deoxyguanosine 5’-monophosphate after hemp oil supplement, indicated that hemp oil could prevents cell damage and gout appearance by regulate the uric acid generation. Chen et al. [126] found the addition of corn oil significantly reduces the serum uric acid content of broilers. The arachidonic acid could be metabolized into different types of eicosanoids by cyclooxygenase, lipoxygenase and cytochrome P450, such as 20-hydroxyeicosatetraenoic acid (20-HETE), thromboxane and prostaglandin E2 (PGE2) [40]. The 20-HETE was a powerful vasoconstrictor and thromboxane was prostaglandin metabolite, and thromboxane B2 (TXB2) was a stable metabolite of thromboxane A2 (TXA2) which promotes coagulation and thrombus formation. Leucene B4 (LTB4) was a metabolite of AA which acts on vascular endothelium to cause inflammatory damage [41]. This study found that excessive added hemp oil significantly up-regulated 20-HETE, which may cause the vascular smooth muscle cells contracted, while lower added hemp oil caused increased LTB4 in the canine serum. The appropriate addition of hemp oil significantly down-regulated the metabolites PGE2, PGD2 and TXB2, which was beneficial to the prevention of anti-inflammatory and cardiovascular diseases. Jing et al. [18] has shown that the PUFA significantly reduces the incidence of cardiovascular disease. Furthermore, the bile acids were mainly derived from the catabolism of cholesterol, and the main ingredients taurocholic acid metabolite deoxycholic acid positive effect on human colon and rectal cancer [31]. The chenodeoxycholic acid was a primary bile acid which could be antibacterial and anti-inflammatory, relieve cough and asthma, and improve immunity [10]. This study found that added appropriate amount of hemp oil reduced taurocholic acid and increased chenodeoxycholic acid, indicated that hemp oil has great potential to prevent cancer, expand the trachea, and improve immunity.

The γ-linolenic and α-linolenic acids play an important role in the body’s immunity, cell growth and anti-inflammation [15], the increased content after hemp oil supplements indicated the enhanced immunity. In addition, the vitamin D3 involved in calcium and bone metabolism and also considered as an essential regulator of immune response [21], the increased content after added appropriate amount of hemp oil which positively prevented rheumatoid arthritis. The ubiquinone as activator of cell respiration and metabolism as well as contributed as antioxidant and nonspecific immune enhancer [34]. Dietary supplement hemp oil significantly enhanced the biosynthesis and oxidative phosphorylation metabolic pathways of ubiquinone and other terpene quinones, increased the content of ubiquinone in canine serum, and enhanced the body’s antioxidant capacity while decreased after excessive supplements.

### 3.3. Correlation among blood metabolomics with biochemical index and nutrient digestibility

**Figure 4.**
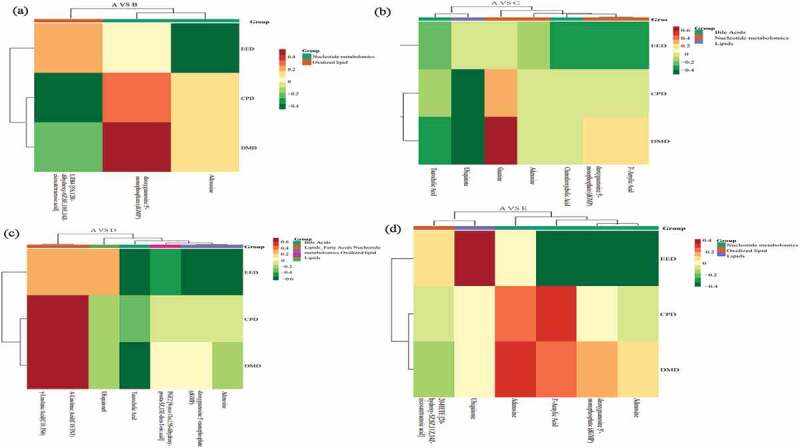
The correlation between metabolomics and nutrient digestibility during control A and treatment B (Figure 4a), C (Figure 4b), D (Figure 4c), and E (Figure 4d).

**Figure 5.**
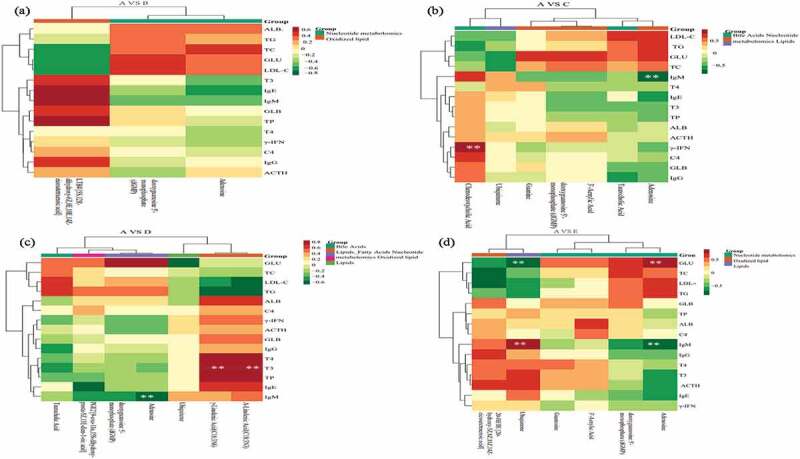
The correlation between metabolomics and blood biochemical index during control A and treatment B (Figure 5a), C (Figure 5b), D (Figure 5c), and E (Figure 5d).

The correlation between metabolomics and nutrient digestibility (Figure 4) showed that between treatment A and B, the 5S,12 R-dihydroxy-6Z,8E,10E and LTB4 positively correlated with EED and negatively correlated with CPD and DMD. The dGMP positively with DMD and CPD, and the adenosine negatively with EED (Figure 4a). Between treatment A and C, the ubiquinone negatively with DMD and CPD while opposite in guanine. The EED negatively with chenodeoxycholic acid, dGMP and 3’-aenylic acid (Figure 4b). Between treatment A and D, the γ-linolenic acid (C18:3 N6) and α-linolenic (C18:3 N3) positively with DMD and CPD, taurocholic acid negatively with EED and DMD, adenosine and dGMP negatively with EED (Figure 4c). Between treatments A and E, EED positively with ubiquinone and negatively with adenosine, dGMP and 3’-aenylic acid (Figure 4d). The correlation between metabolomics and blood biochemical index is shown in Figure 5. Between treatment A and B, the LTB4 positively correlated with TP, IgM, IgG, IgE and negatively correlated with TC, GLU and LDLC. The dGMP positively with GLU and LDLC, and the adenosine positively related with TC and negatively with IgE (Figure 5a). Between treatment A and C, the chenodeoxycholic acid significant positively related to γ-IFN. The adenosine significantly negatively with IgM and positively with TC, GLU and LDLC. The GLU positively with guanine, dGMP, 3’-aenylic acid and adenosine (Figure 5b). Between treatments A and D, taurocholic acid was positively related to TG and LDLC, adenosine significantly negative with IgM and positively with GLU. The γ-linolenic acid (C18:3 N6) and α-linolenic acid (C18:3 N3) were significant positively with T3, T4 and TP, negatively with TG (Figure 5c). Between treatments A and E, ubiquinone significant positively with IgM and negatively with GLU while opposite in adenosine (Figure 5d).

**Figure 6.**
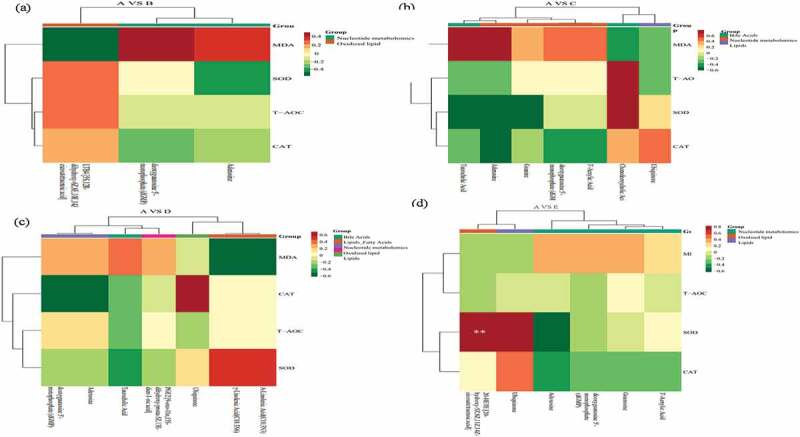
The correlation between metabolomics and blood antioxidant indicators during control A and treatment B (Figure 6a), C (Figure 6b), D (Figure 6c), and E (Figure 6d). A: dietary supplements 0% hemp oil in teddy dog (as control), B: dietary supplements 0.5% hemp oil in teddy dog, C: dietary supplements 1% hemp oil in teddy dog, D: dietary supplements 2% hemp oil in teddy dog and E: dietary supplements 4% hemp oil in teddy dog.

The correlation between metabolomics and blood antioxidant indicators was presented in Figure 6. Between treatment A and B, the LTB4 positively correlated with SOD, T-AOC, CAT and negatively correlated with MDA. The dGMP was positively with MDA and negatively with CAT. The adenosine positively related to MDA and negatively with SOD (Figure 6a). Between treatments A and C, the chenodeoxycholic acid was positively related with SOD, T-AOC and negatively with MDA. The adenosine negatively with SOD, CAT and positively with MDA (Figure 6b). Between treatment A and D, γ-linolenic acid (C18:3 N6) and α-linolenic acid (C18:3 N3) positively with SOD, negatively with MDA. The CAT positively with ubiquinone and negatively with adenosine and dGMP (Figure 6c). Between treatment A and E, SOD significant positively with ubiquinone and LTB4, and negatively with adenosine (Figure 6d). The adenosine and blood sugar showed a significant positive correlation after 4% hemp oil supplement, while 1% to 4% hemp oil addition significantly down-regulated adenosine, thus less adenosine bound to A2A receptors which cannot inhibit the proliferation, migration, and survival of T cells, further occurs humoral immunity and the IgM content increased, finally adenosine and IgM showed a significant negative correlation [35]. The hemp oil reduced blood sugar and probably because of decreased adenosine which relieves inflammation. In addition, lower chenodeoxycholic acid significantly improves the phagocytic function of mononuclear macrophages, while the higher content causes immunosuppressive effect [42]. As an important cytokine in cellular immunity, interferon-γ mainly promotes the proliferation and differentiation of T lymphocytes, activates monocyte-macrophages, and improves cellular immune function [38]. In this study, the addition of hemp oil enhanced the body’s immune function which might be caused by the combined action of chenodeoxycholic acid and γ-interferon. The γ-linolenic acid and α-linolenic acid were significant positive related to triiodothyronine which considered as an active substance and could be realized thyroxine physiology, might be possible reasons for immunity enhancement [43]. The ubiquinone was significantly negative correlated with blood sugar and positively correlated with IgM. Studies have found that ubiquinone promotes the secretion and synthesis of insulin and reduced blood sugar as well as promotes antibody production and improves immunity capacity. Finally, hemp oil supplementation active in regulates the association of blood metabolites and nutrient digestion, favorable to immunity and digestion improve as well as disease relief related metabolism, 1–2% hemp oil was recommended dosage for teddy dog.

## 4. Conclusion

The dietary supplements 1–2% hemp oil in teddy dogs significantly improved the immunity status and antioxidant capacity, enhanced apparent nutrient digestibility while suppressed in 4% hemp oil dietary supplementation. additionally, hemp oil altered blood biochemical index and metabolism and a total of 440 metabolites were detected. Hemp oil increased the content and types of beneficial metabolites, improved the immunity capacity and regulated lipid metabolism. Notably, 1–2% hemp oil demonstrated better performance and recommended for teddy dog diet.

## Novelty Statement

This study evaluated the influence of dietary supplements hemp oil on apparent nutrient digestibility, blood biochemical parameters and metabolomics of teddy dogs. The result suggested that dietary supplements 1-2% hemp oil in teddy dog significantly improved the immunity status and antioxidant capacity, enhanced apparent nutrient digestibility while suppressed in 4% hemp oil dietary supplementation. Furthermore, hemp oil altered blood biochemical index and metabolism, increased the content and types of beneficial metabolites and regulated lipid metabolism. Notably, 1-2% hemp oil demonstrated better performance and recommended for teddy dog diet.
